# Dopamine and cAMP‐regulated phosphoprotein 32kDa (DARPP‐32), protein phosphatase‐1 and cyclin‐dependent kinase 5 expression in ovarian cancer

**DOI:** 10.1111/jcmm.15553

**Published:** 2020-06-25

**Authors:** Stewart G. Martin, Siwei Zhang, Song Yang, Behnaz Saidy, Suha Deen, Sarah J. Storr

**Affiliations:** ^1^ Nottingham Breast Cancer Research Centre Division of Cancer and Stem Cells School of Medicine University of Nottingham Biodiscovery Institute Nottingham UK; ^2^ Pathology Group London UK

**Keywords:** Cdk5, DARPP‐32, ovarian cancer, PKA, PP1, prognosis

## Abstract

Dopamine and cyclic‐AMP activated phosphoprotein Mr32kDa (DARPP‐32) is a central signalling protein in neurotransmission. Following DARPP‐32 phosphorylation by protein kinase A (PKA), DARPP‐32 becomes a potent protein phosphatase 1 (PP1) inhibitor. DARPP‐32 can itself inhibit PKA following DARPP‐32 phosphorylation by cyclin‐dependent kinase 5 (Cdk5). Increasing evidence indicates a role for DARPP‐32 and its associated signalling pathways in cancer; however, its role in ovarian cancer remains unclear. Using immunohistochemistry, expression of DARPP‐32, PP1 and Cdk5 was determined in a large cohort of primary tumours from ovarian cancer patients (n = 428, 445 and 434 respectively) to evaluate associations between clinical outcome and clinicopathological criteria. Low cytoplasmic and nuclear DARPP‐32 expression was associated with shorter patient overall survival and progression‐free survival (*P* = .001, .001, .004 and .037 respectively). Low nuclear and cytoplasmic DARPP‐32 expression remained significantly associated with overall survival in multivariate Cox regression (*P* = .045, hazard ratio (HR) = 0.734, 95% confidence interval (CI) = 0.542‐0.993 and *P* = .001, HR = 0.494, 95% CI = 0.325‐0.749, respectively). High cytoplasmic and nuclear PP1 expression was associated with shorter patient overall survival and high cytoplasmic PP1 expression with shorter progression‐free survival (*P* = .005, .033, and .037, respectively). High Cdk5 expression was associated with shorter progression‐free survival (*P* = .006). These data suggest a role for DARPP‐32 and associated signalling kinases as prognostic markers with clinical utility in ovarian cancer.

## 1 INTRODUCTION

Dopamine and cyclic‐AMP (cAMP)‐regulated phosphoprotein Mr32kDa (DARPP‐32) is a target of dopamine and cAMP and is found enriched in dopaminoceptive nerve terminals where it acts as a central signalling molecule. Dependent upon its phosphorylation state, DARPP‐32 has been shown to act as a phosphatase inhibitor or a kinase. DARPP‐32 acts as an inhibitor of protein phosphatase 1 (PP1) and protein kinase A (PKA).^1^ Phosphorylation at Threonine (Thr)‐34 by PKA converts DARPP‐32 to a potent inhibitor of PP1, whereas phosphorylation at Thr‐75 by cyclin‐dependent kinase 5 (Cdk5) or cell division cycle (cdc)2 converts DARPP‐32 into a PKA inhibitor.^2^ There is further complexity in this signalling, with phosphorylation of DARPP‐32 Serine (Ser)‐137 by casein kinase 1 (CK1) able to prevent dephosphorylation of Thr‐34.^3^ A truncated DARPP‐32 splice variant, t‐DARPP, exists that has an alternate translational start in exon 2. The t‐DARPP splice variant lacks the Thr‐34 phosphorylation site and consequently, it is unable to inhibit PP1. DARPP‐32 and t‐DARPP expression, including DARPP‐32 to t‐DARPP expression ratios, have been linked with patient survival in a number of tumour types, including breast, non‐small cell lung, gastric colorectal and oesophageal cancers.^4^, ^5^, ^6^, ^7^, ^8^, ^9^ Expression of t‐DARPP has been shown in gastric, non‐small cell lung and breast cancer, amongst other tumour types.^4^, ^6^, ^10^

DARPP‐32 acts as a central signalling protein in neurotransmission through its inhibition of PP1 and PKA. PP1 is a heterotrimer and acts as a Ser/Thr phosphatase in many important cellular functions, including mitosis.^11^ In cancer, amplification of the *PPP1CA* gene causes aberrant activation of mitogen‐activated protein kinase (MAPK) in prostate cancer,^12^ and in glioblastoma, nuclear PP1A expression is associated with survival of patients with p53 expressing tumours.^13^

DARPP‐32 phosphorylation of Thr‐75 by Cdk5 allows DARPP‐32 to function as a PKA inhibitor. Cdk5 is considered a neuronal serine/threonine kinase that is predominantly activated by p35 or p39; its function has been implicated in a number of tumourigenic pathways, with a number of important substrates in addition to DARPP‐32, including p53 and AKT (reviewed in Ref. [14]). Studies in a number of tumour types have demonstrated that high Cdk5 expression is associated with clinicopathological criteria associated with poor prognosis, and in some cases, shortened disease‐specific survival itself^15^, ^16^, ^17^; however, the reverse has been observed in gastric cancer.^18^, ^19^

There is increasing evidence that DARPP‐32, PP1 and Cdk5 have a role in various tumour types; however, expression of DARPP‐32 and PP1 has not previously been described in ovarian cancer, although DARPP‐32 has been implicated in follicular development.^20^ In ovarian cancer, in vitro studies have indicated a role for Cdk5 in paclitaxel sensitivity,^21^ DNA damage response,^22^ mitosis^23^ and AKT activation.^24^ Ovarian cancer is the seventh most common cancer in woman globally, and five‐year survival is around 45%.^25^ Treatment for ovarian cancer principally consists of surgery and platinum‐based chemotherapy. The current study sought to determine DARPP‐32, PP1 and Cdk5 expression in ovarian cancer and determine their relationships with patient survival.

## 2 MATERIALS AND METHODS

### 2.1 Patient cohorts

Patients received treatment at Nottingham University Hospitals between 1991 and 2011. Progression‐free survival was defined as the length between the start of treatment and clinical identification of recurrence or last follow‐up date. Overall survival was defined as the length between the start of treatment and date of death or last follow‐up date. Median follow‐up was 100 months determined using the reverse Kaplan‐Meier method, and clinicopathological characteristics of the cohort are shown in Table 1. Clinicopathological information available included patient age, Figo stage, tumour grade, residual disease, response to chemotherapy and histological subtype. Age was categorized based on the median age of the patient cohort. Suboptimal debulking was classified as residual disease of >2 cm. Data on chemotherapy resistance were recorded according to the Gynaecological Oncology Group (COG) as refractory (not responding to chemotherapy), resistant (an initial response to chemotherapy with recurrence within 6 months) or sensitive (either no recurrence, or recurrence after 6 months). Ethical approval was obtained from Derbyshire Ethics Committee (07/H0401/156), This study is reported in accordance to REMARK criteria.^26^

**TABLE 1:** Associations between the cytoplasmic and nuclear expression of DARPP‐32, PP1 and Cdk5 determined using immunohistochemistry with clinicopathological variables. The *P* values are resultant from Pearson's *χ* ^2^ test of association, and significant values (*P* ≤ .05) are highlighted in bold

	DARPP‐32						PP1						Cdk5					
	Cytoplasmic expression			Nuclear expression			Cytoplasmic expression			Nuclear expression			Cytoplasmic expression			Nuclear expression		
	Low	High	*P*	Low	High	*P*	Low	High	*P*	Low	High	*P*	Low	High	*P*	Low	High	*P*
Patient age																		
62 years and below	159 (37.1%)	33 (7.7%)	.947	119 (27.9%)	73 (17.1%)	.412	164 (36.9%)	35 (7.9%)	.465	97 (21.8%)	101 (22.7%)	.115	118 (27.3%)	80 (18.5%)	.283	68 (15.7%)	130 (30.0%)	.302
Older than 62	196 (45.9%)	40 (9.4%)		154 (36.2%)	80 (18.8%)		196 (44.0%)	50 (11.2%)		139 (31.3%)	107 (24.1%)		128 (29.6%)	107 (24.7%)		92 (21.2%)	143 (33.0%)	
Figo stage																		
1	122 (28.9%)	26 (6.2%)	.926	88 (21.0%)	60 (14.3%)	.258	147 (33.4%)	13 (3.0%)	**<.001**	96 (21.9%)	63 (14.4%)	.147	101 (23.8%)	52 (12.1%)	**.004**	74 (17.3%)	79 (18.5%)	**.002**
2	41 (9.7%)	8 (1.9%)		32 (7.6%)	16 (3.8%)		37 (8.4%)	12 (2.7%)		23 (5.2%)	26 (5.9%)		27 (6.3%)	18 (4.2%)		18 (4.2%)	27 (6.3%)	
3	160 (37.9%)	34 (8.1%)		126 (30.0%)	67 (16.0%)		148 (33.6%)	53 (12.0%)		104 (23.7%)	97 (22.1%)		96 (22.4%)	106 (24.8%)		57 (13.3%)	145 (33.9%)	
4	27 (6.4%)	4 (0.9%)		24 (5.7%)	7 (1.7%)		23 (5.2%)	7 (1.6%)		13 (3.0%)	17 (3.9%)		18 (4.2)	10 (2.3%)		10 (2.3%)	18 (4.2%)	
Tumour grade																		
1	25 (6.1%)	10 (2.3%)	**.041**	16 (3.8%)	19 (4.5%)	**<.001**	33 (7.4%)	2 (0.4%)	**.002**	24 (5.4%)	11 (2.5%)	.115	26 (6.0%)	11 (2.5%)	.151	19 (4.4%)	18 (4.1%)	**.045**
2	24 (11.2%)	15 (3.5%)		27 (6.3%)	36 (8.5%)		59 (13.3%)	5 (1.1%)		32 (7.2%)	32 (7.2%)		38 (8.8%)	25 (5.8%)		28 (6.5%)	35 (8.1%)	
3	281 (65.7%)	48 (11.2%)		230 (54.0%)	98 (23.0%)		268 (60.2%)	78 (17.5%)		180 (40.5%)	165 (37.2%)		182 (41.9%)	152 (35.0%)		113 (26.0%)	221 (50.9%)	
Residual disease																		
No residual tumour	183 (48.4%)	42 (11.1%)	.232	135 (35.9%)	90 (23.9%)	.137	217 (54.7%)	28 (7.1%)	**<.001**	147 (37.1%)	97 (24.5%)	**.010**	149 (38.9%)	87 (22.7%)	**.001**	101 (26.4%)	135 (35.2%)	**.017**
Residual tumour < 2 cm	35 (9.3%)	11 (2.9%)		14 (9.0%)	11 (9.2%)		35 (8.8%)	13 (3.3%)		19 (4.8%)	29 (7.3%)		20 (5.2%)	27 (7.0%)		12 (3.1%)	35 (9.1%)	
Residual tumour > 2 cm	93 (24.6%)	14 (3.7%)		68 (18.1%)	38 (10.1%)		72 (18.1%)	32 (8.1%)		50 (12.6%)	54 (13.6%)		44 (11.5%)	56 (14.6%)		30 (7.8%)	70 (18.3%)	
Response to chemotherapy																		
Refractory	24 (8.8%)	5 (1.8%)	.360	23 (8.5%)	6 (2.2%)	.210	29 (10.1%)	5 (1.7%)	.123	22 (7.7%)	12 (4.2%)	.43	16 (5.7%)	16 (5.7%)	.704	10 (3.5%)	22 (7.8%)	.787
Relapsed within 6 months	18 (6.6%)	1 (0.4%)		13 (4.8%)	6 (2.2%)		20 (6.9%)	1 (0.3%)		12 (4.2%)	9 (3.1%)		9 (3.2%)	11 (3.9%)		7 (2.5%)	13 (4.6%)	
Relapsed after 6 months	185 (67.5%)	41 (15.0%)		141 (51.8%)	80 (18.8%)		182 (63.2%)	51 (17.7%)		123 (42.9%)	109 (38.0%)		124 (44.0%)	106 (37.6%)		86 (30.5%)	144 (51.1%)	
Histology																		
High‐grade serous carcinoma	225 (52.6%)	41 (9.6%)	**.028**	180 (42.3%)	85 (20.0%)	**<.001**	196 (44.0%)	78 (17.5%)	**<.001**	132 (29.7%)	141 (31.8%)	.182	127 (29.3%)	139 (32.0%)	**<.001**	74 (17.1%)	192 (44.2%)	**<.001**
Mucinous	33 (7.7%)	9 (2.1%)		20 (4.7%)	22 (5.2%)		40 (9.0%)	1 (0.2%)		23 (5.2%)	18 (4.1%)		29 (6.7%)	15 (3.5%)		22 (5.1%)	22 (5.1%)	
Endometrioid	36 (8.4%)	16 (3.7%)		22 (5.2%)	30 (7.0%)		48 (10.8%)	3 (0.7%)		33 (7.4%)	18 (4.1%)		34 (7.8%)	20 (4.6%)		28 (65%)	26 (6.0%)	
Clear cell carcinoma	37 (8.6%)	1 (0.2%)		37 (8.7%)	1 (0.2%)		48 (10.8%)	0 (0.0%)		29 (6.5%)	19 (22.5%)		38 (8.8%)	3 (0.7%)		24 (5.5%)	17 (3.9%)	
Low‐grade serous carcinoma	17 (4.0%)	4 (0.9%)		11 (2.6%)	9 (2.1%)		18 (4.0%)	2 (0.4%)		13 (2.9%)	7 (1.6%)		10 (2.3%)	8 (1.8%)		8 (1.8%)	10 (2.3%)	
Borderline serous carcinoma	6 (1.4%)	2 (0.5%)		2 (0.5%)	6 (1.4%)		10 (2.2%)	1 (0.2%)		6 (1.4%)	5 (1.1%)		8 (1.8%)	3 (0.7%)		4 (0.9%)	7 (1.6%)	
Borderline mucinous carcinoma	1 (0.2%)	0 (0.0%)		0 (0.0%)	0 (0.0%)		0 (0.0%)	0 (0.0%)		0 (0.0%)	0 (0.0%)		0 (0.0%)	0 (0.0%)		0 (0.0%)	0 (0.0%)	

### 2.2 Immunohistochemistry

Immunohistochemical staining was conducted using a Novolink Polymer Detection kit (Leica) on a tissue microarray comprised of single 0.6mm cores from 575 ovarian tumours taken from a representative area as assessed by a specialist ovarian cancer histopathologist; the use of which has been described previously.^27^, ^28^, ^29^ Staining was conducted according to manufacturers’ instructions and has been described previously.^30^ Briefly, tissue was deparaffinized in xylene, rehydrated in ethanol then water and heated in a microwave for 10 minutes at 750 W followed by 10 minutes at 450 W in 0.01 mol L^−1^ sodium citrate buffer (pH6.0). Novolink Peroxidase Block was incubated on the tissue, washed with Tris‐buffered saline (TBS), followed by incubation with Novolink Protein Block solution. Rabbit polyclonal anti‐DARPP‐32 (Abcam ab40801) diluted 1:500, anti‐Cdk5 (Cell Signalling Technology 1H3) diluted 1:500 and anti‐PP1 (Life Technologies 10C6‐3) diluted 1:50 were used as the primary antibodies; each was incubated on tissue for one hour at room temperature; with antibody specificity confirmed prior to use in immunohistochemistry by Western blotting. Tissue was washed with TBS prior to incubation with Novolink Post Primary solution, which was subsequently washed with TBS and then incubation with Novolink Polymer solution. 3,3’ diaminobenzidine was used as the chromogenic substrate to develop immunohistochemical reactions and tissue was counterstained with haematoxylin. Positive and negative controls were included with each staining run and were comprised of breast tumour composite sections comprising grade 1 and 2 early stage invasive tumours; negative controls had primary antibody omitted from each staining run.

Slides were scanned using a Nanozoomer Digital Pathology Scanner (Hamamatsu Photonics), and staining was assessed at 200× magnification. Staining in the cytoplasm was assessed using a semi‐quantitative immunohistochemical H score, where staining intensity within tumour cells was assessed as none (0), weak (1), medium (2) or strong (3) over the percentage area of each staining intensity. Staining in the nucleus was examined in a semi‐quantitative manner, where the percentage of tumour cells that demonstrated any staining intensity was assessed. Greater than 30% of cores for each TMA were double assessed, with both assessors blinded to clinical outcome and each other's scores.

### 2.3 Statistical analyses

Statistical analysis was performed using IBM SPSS Statistics (version 26). Cases were stratified based on overall survival using X‐Tile software.^31^ All differences were deemed statistically significant at the level of *P* ≤ .05. The Pearson *χ* ^2^ test of association was used to determine the relationship between categorized protein expression and clinicopathological variables. Survival curves were plotted according to the Kaplan‐Meier method with significance determined using the log‐rank test. Cox proportional hazards regression model was used for multivariate survival analysis. The primary end point of this study was to determine whether the expression of protein was associated with overall survival of ovarian cancer patients.

## 3 RESULTS

### 3.1 DARPP‐32, PP1 and Cdk5 protein staining location and frequency

**FIGURE 1:**
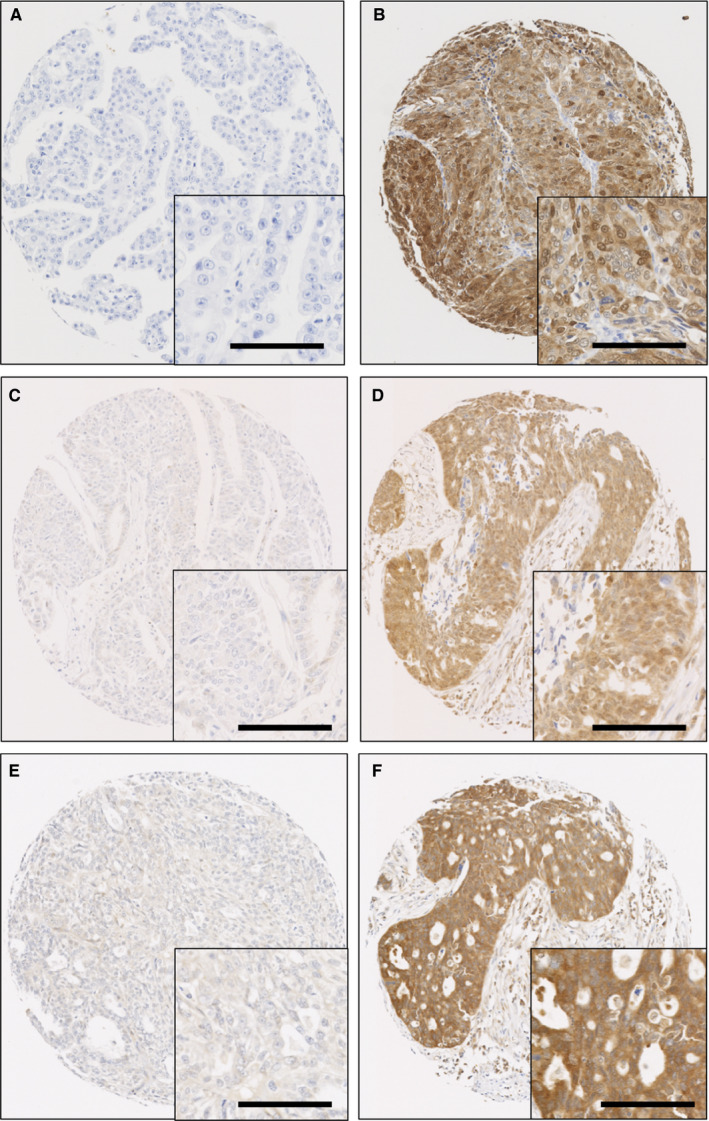
Representative immunohistochemical staining of DARPP‐32, PP1 and Cdk5. Low expression of DARPP‐32 (A), high expression of DARPP‐32 (B); low expression of PP1 (C), high expression of PP1 (D). Low expression of Cdk5 (E) and high expression of Cdk5 (F) are shown. 100× magnification is shown, with a 200× magnification inset box; scale bar shows 100 µM

Protein expression was assessed in ovarian cancer tissue; 428, 445 and 434 patients were available for assessment for DARPP‐32, PP1 and Cdk5, respectively. Different numbers of patient specimens were available due to core attrition and/or insufficient tumour available to score. Nuclear and cytoplasmic expression of DARPP‐32, PP1 and Cdk5 was observed and staining varied from weak to intense, with heterogeneity observed between adjacent tumour cells. Representative photomicrographs are shown in Figure 1.

Nuclear DARPP‐32 expression had a median H‐score of 10 and ranged from 0 to 100 with cytoplasmic DARPP‐32 expression having a median H‐score of 65 and ranging from 0 to 300. X‐tile was used to generate cut points based on overall survival; nuclear DARPP‐32 expression had a cut point of 30 and 64.1% of cases (227/426) demonstrated low expression. Cytoplasmic DARPP‐32 expression had a cut point of 175 and 82.9% of cases (355/428) had low expression. Nuclear PP1 expression had a median H‐score of 60 and ranged from 0 to 100 and cytoplasmic PP1 expression had a median H‐score of 90 and ranged from 0 to 280. Nuclear PP1 had a cut point of 60 and 53.2% of cases (236/444) demonstrated low expression; cytoplasmic PP1 had a cut point of 140 and 80.9% of cases (360/445) demonstrated low expression. Nuclear Cdk5 expression had a median H‐score of 10 and ranged from 0‐100 and cytoplasmic Cdk5 expression had a median H‐score of 120 and ranged from 0‐270. Nuclear Cdk5 had a cut point of 10 and 36.9% of cases (160/434) demonstrated low expression; cytoplasmic Cdk5 had a cut point of 130 and 56.7% of cases (246/434) demonstrated low expression.

### 3.2 Relationship between DARPP‐32, PP1 and Cdk5

The relationship between DARPP‐32, PP1 and Cdk5 cytoplasmic and nuclear expression was explored using the Spearman rank correlation coefficient. DARPP‐32 cytoplasmic expression was significantly correlated with DARPP‐32 nuclear expression (*r* ^2^ = .858, *P* < .001). PP1 cytoplasmic expression was significantly correlated with PP1 nuclear expression (*r* ^2^ = .487, *P* < .001). Cdk5 cytoplasmic expression was significantly correlated with Cdk5 nuclear expression (*r* ^2^ = .615, *P* < .001). All of these correlations indicate a strong biological relationship between nuclear and cytoplasmic expression of each protein that has not been assessed further within this study.

DARPP‐32 cytoplasmic expression was correlated with PP1 cytoplasmic (*r* ^2^ = .285, *P* < .001), PP1 nuclear (*r* ^2^ = .147, *P* < .001), Cdk5 cytoplasmic (*r* ^2^ = .271, *P* < .001) and Cdk5 nuclear expression (*r* ^2^ = .200, *P* < .001). DARPP‐32 nuclear expression was correlated with PP1 cytoplasmic (*r* ^2^ = .146, *P* < .001) and Cdk5 cytoplasmic expression (*r* ^2^ = .188, *P* < .001), but not PP1 nuclear (*r* ^2^ = .081, *P* = .110) and Cdk5 nuclear expression (*r* ^2^ = .100, *P* = .51). Cytoplasmic PP1 expression was correlated with Cdk5 cytoplasmic (*r* ^2^ = .610, *P* < .001) and nuclear expression (*r* ^2^ = .357, *P* < .001). Nuclear PP1 expression was correlated with Cdk5 cytoplasmic (*r* ^2^ = .304, *P* < .001) and Cdk5 nuclear expression (*r* ^2^ = .440, *P* < .001).

### 3.3 Relationship between DARPP‐32, PP1 and Cdk5 protein expression and clinicopathological variables

Pearson's chi‐squared tests were performed to evaluate the relationships between DARPP‐32, PP1 and Cdk5 expression with available clinicopathological criteria (Table 1). Low nuclear DARPP‐32 expression was associated with grade 3 tumours (*χ* ^2^ = 22.660, *df* = 2, *P* < .001) and tumour histology (*χ* ^2^ = 42.236, *df* = 6, *P* < .001; Table 1). Low cytoplasmic DARPP‐32 expression was associated with grade 2 tumours (*χ* ^2^ = 6.371, *df* = 2, *P* = .041) and tumour histology (*χ* ^2^ = 14.197, *df* = 6, *P* = .028; Table 1). Low nuclear expression of PP1 was associated with absence of residual disease (*χ* ^2^ = 9.287, *df* = 2, *P* = .010; Table 1). Low cytoplasmic expression of PP1 was associated with lower Figo stage (*χ* ^2^ = 20.422, *df* = 3, *P* < .001), lower tumour grade (*χ* ^2^ = 11.990, *df* = 2, *P* = .002), absence of residual disease (*χ* ^2^ = 20.949, *df* = 2, *P* < .001) and tumour histology (*χ* ^2^ = 41.807, *df* = 5, *P* < .001; Table 1). Low nuclear Cdk5 expression was associated with lower Figo stage (*χ* ^2^ = 15.327, *df* = 3, *P* = .002), lower tumour grade (*χ* ^2^ = 6.211, *df* = 2, *P* = .045), absence of residual disease (*χ* ^2^ = 8.107, *df* = 2, *P* = .017) and tumour histology (*χ* ^2^ = 26.522, *df* = 6, *P* < .001; Table 1). Low cytoplasmic Cdk5 expression was associated with lower Figo stage (*χ* ^2^ = 13.172, *df* = 3, *P* = .004) tumour histology (*χ* ^2^ = 33.852, *df* = 6, *P* < .001) and the absence of residual disease (*χ* ^2^ = 14.121, *df* = 2, *P* = .001; Table 1).

### 3.4 Association between DARPP‐32, PP1 and Cdk5 protein expression and overall survival

**FIGURE 2:**
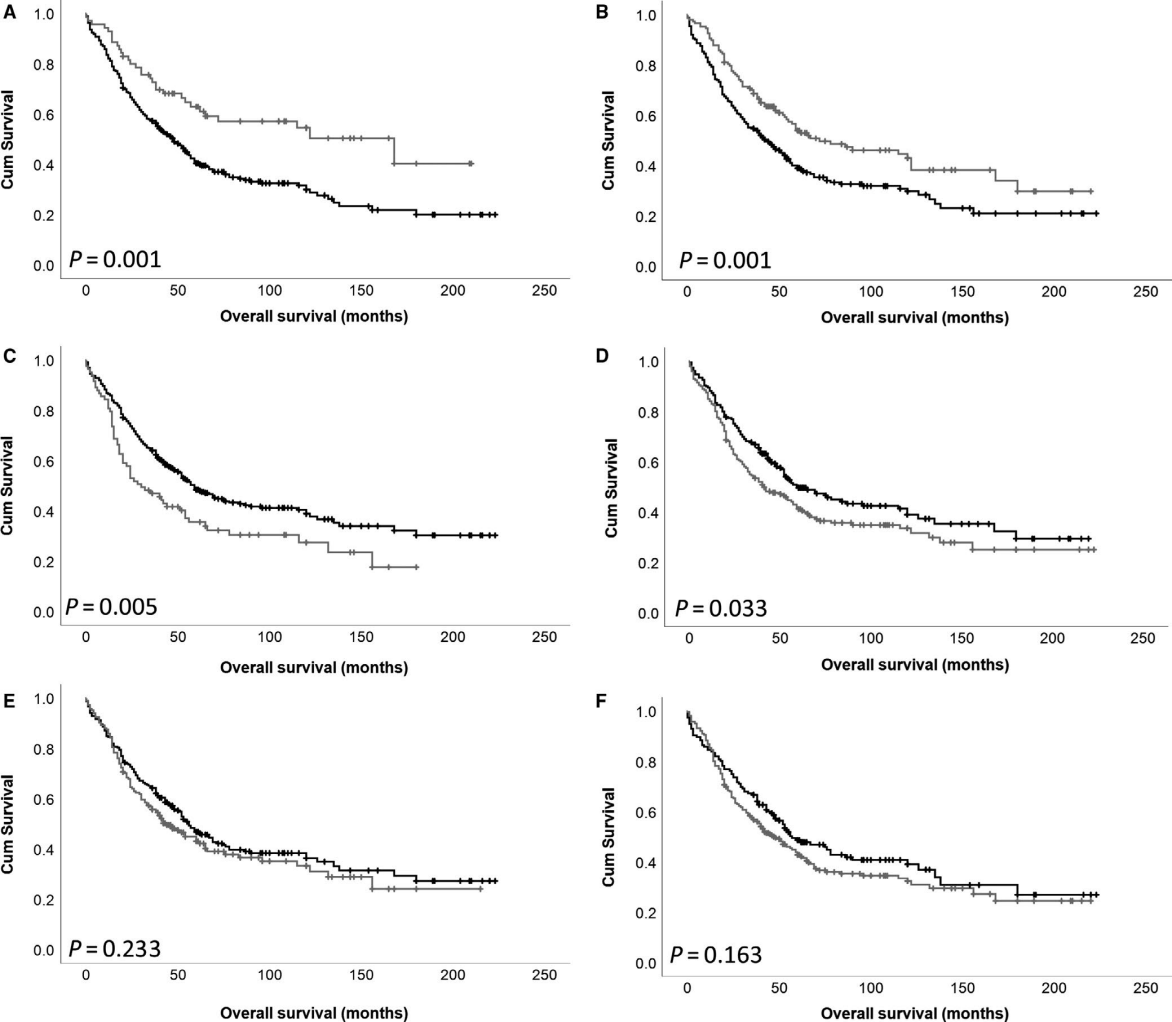
Kaplan‐Meier analysis of ovarian cancer overall survival showing the impact of low (black line) and high (grey line) DARPP‐32 expression within the cytoplasm (A) and nucleus (B), PP1 expression within the cytoplasm (C) and nucleus (D) and Cdk5 expression within the cytoplasm (E) and nucleus (F). Significance was determined using the log‐rank test

Low DARPP‐32 expression in the cytoplasm and in the nucleus was associated with adverse survival (both *P* = .001; Figure 2A,B). Low nuclear DARPP‐32 expression remained significantly associated with survival in multivariate Cox regression (*P* = .045, hazard ratio (HR) = 0.734, 95% confidence interval (CI) = 0.542‐0.993), when Figo stage, residual disease, tumour grade, tumour histology and median patient age were included (all with individual Kaplan‐Meier statistics of *P* = .001 or below). Low cytoplasmic DARPP‐32 expression also remained significant in multivariate Cox regression (*P* = .001, HR = 0.494, 95% CI = 0.325‐0.749) when the same variables were included. Low cytoplasmic and nuclear PP1 expression was associated with improved survival (*P* = .005 and .033) (Figure 2C,D); low cytoplasmic and nuclear PP1 expression was not associated with survival in multivariate Cox regression (*P* = .434, HR = 0.874, 95% CI = 0.624‐1.225 and *P* = .245, HR = 1.178, 95% CI = 0.894‐1.552). Cdk5 cytoplasmic and nuclear expression was not associated with patient survival (Figure 2E,F).

### 3.5 Association between DARPP‐32, PP1 and Cdk5 protein expression and progression‐free survival

**FIGURE 3:**
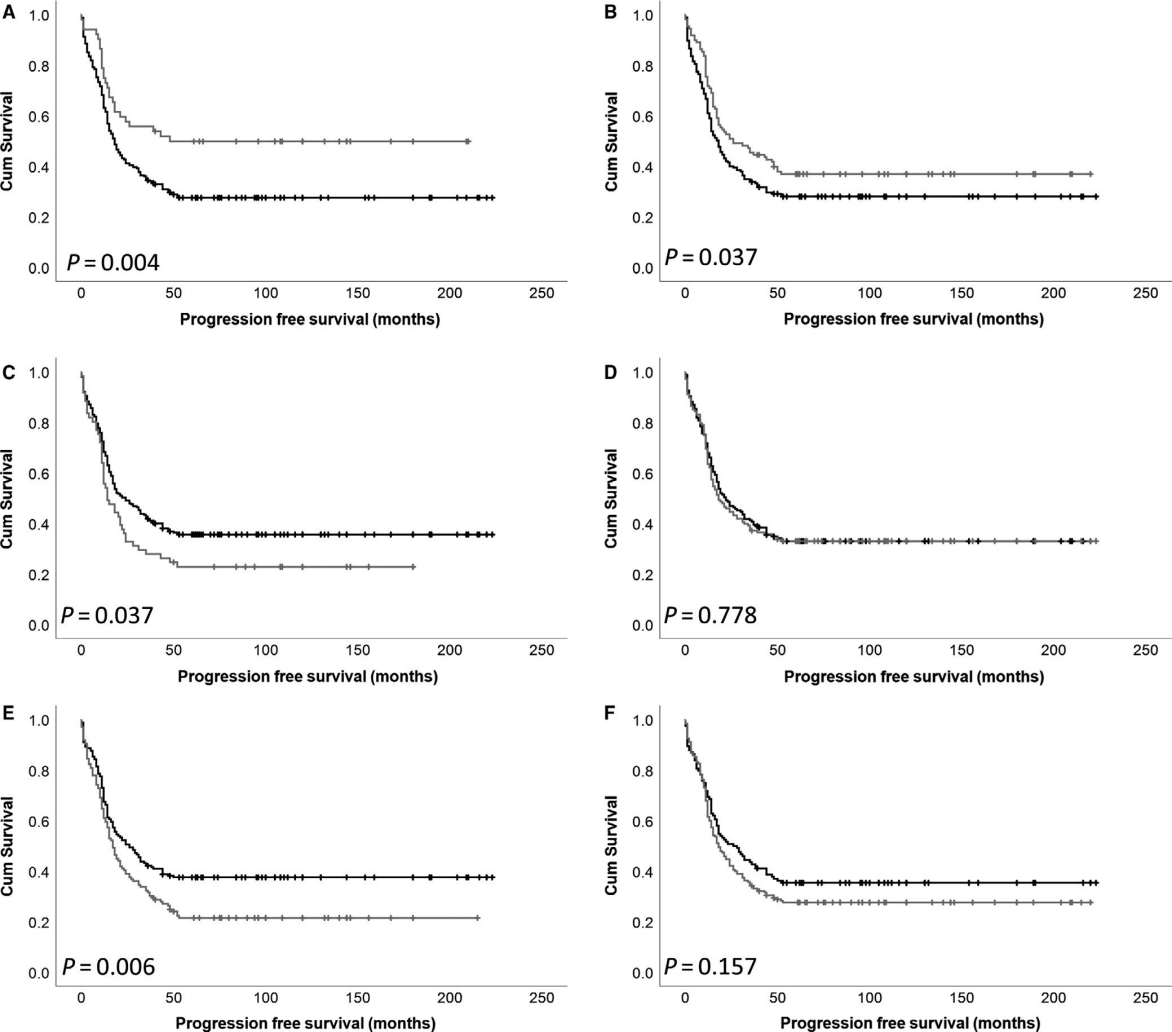
Kaplan‐Meier analysis of ovarian progression‐free survival showing the impact of low (black line) and high (grey line) DARPP‐32 expression within the cytoplasm (A) and nucleus (B), PP1 expression within the cytoplasm (C) and nucleus (D) and Cdk5 expression within the cytoplasm (E) and nucleus (F). Significance was determined using the log‐rank test

Low DARPP‐32 expression in the cytoplasm and in the nucleus was also associated with shorter progression‐free survival (*P* = .004 and .037, respectively) (Figure 3A,B). Low cytoplasmic DARPP‐32 expression remained significantly associated with progression‐free survival in multivariate Cox regression (*P* = .006, HR = 0.546, 95% CI = 0.355‐0.840), when Figo stage, residual disease, tumour grade, tumour histology and median patient age were included (all with individual Kaplan‐Meier statistics of *P* = .001 or below). Low PP1 expression in the cytoplasm, but not the nucleus was associated with improved progression‐free survival (*P* = .037; Figure 3C,D); PP1 cytoplasmic expression was not associated with progression‐free survival in multivariate Cox regression (*P* = .143, HR = 0.772, 95% CI = 0.545‐1.092). Low cytoplasmic Cdk5 expression, but not nuclear expression, was associated with improved progression‐free survival (*P* = .006; Figure 3E,F); Cdk5 cytoplasmic expression was not associated with progression‐free survival in multivariate Cox regression (*P* = .962, HR = 1.007, 95% CI = 0.750‐1.352).

### 3.6 Association between DARPP‐32, PP1 and Cdk5 protein expression and overall survival in high‐grade serous carcinomas

Expression levels of DARPP‐32, PP1 and Cdk5 were assessed in the high‐grade serous carcinoma histological subtype. In this histological group, DARPP‐32 cytoplasmic and nuclear expression, PP1 cytoplasmic and nuclear expression and Cdk5 cytoplasmic and nuclear expression were not associated with overall survival (*P* = .073, .098, .518, .294, .815 and .497, respectively). Other histological subtypes were not individually assessed as they had limited events available for assessment.

### 3.7 Association between DARPP‐32, PP1 and Cdk5 protein expression combinations and overall survival

**FIGURE 4:**
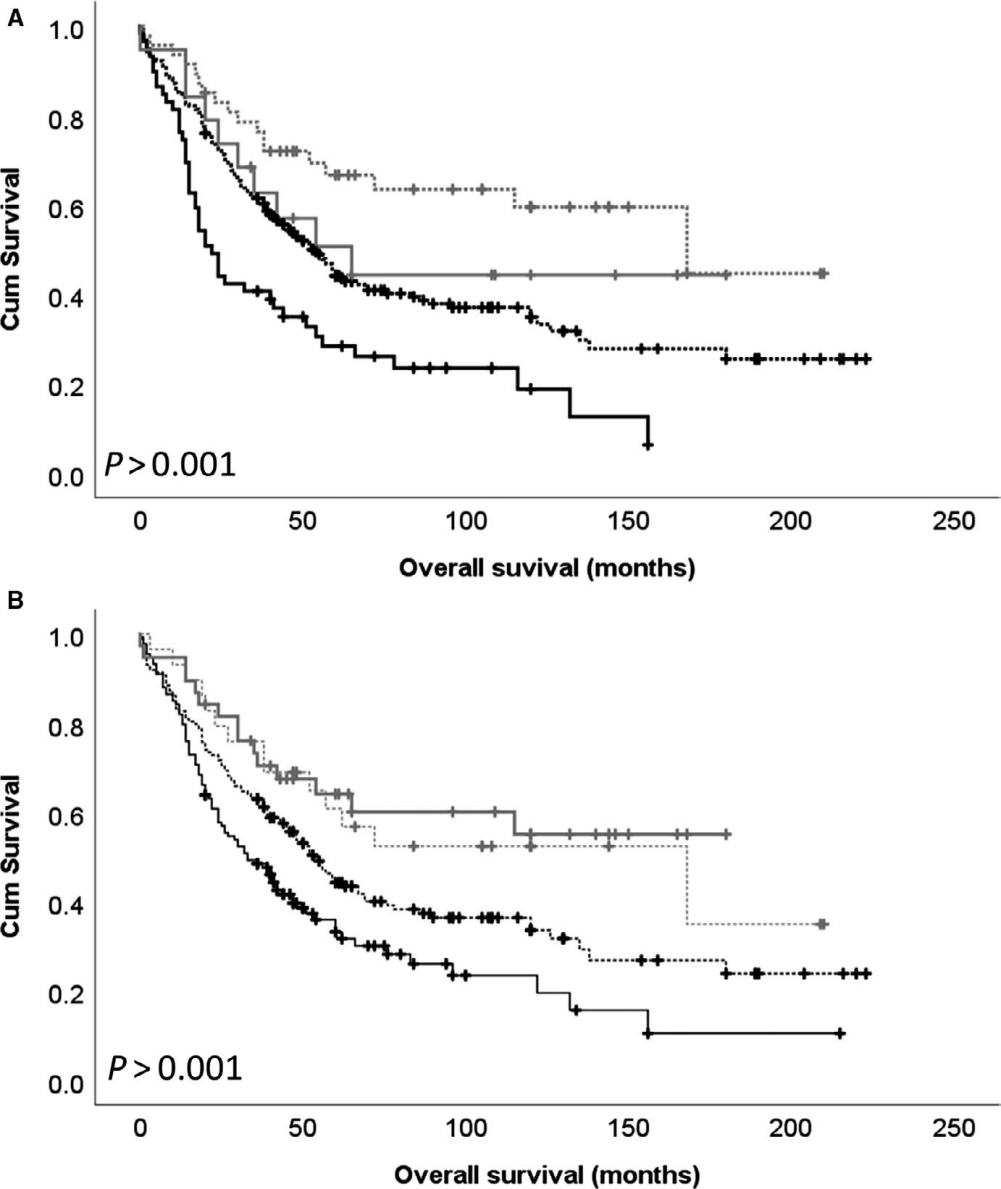
Kaplan‐Meier analysis of ovarian overall survival showing the impact of the combination of cytoplasmic DARPP‐32 expression with cytoplasmic PP1 expression (A) and cytoplasmic Cdk5 expression (B). Low DARPP‐32 expression is indicated by a black line and high DARPP‐32 expression is indicated by a grey line. The dashed line indicates low PP1 or Cdk5 expression, and the solid line indicates high PP1 or Cdk5 expression. Significance was determined using the log‐rank test

High and low cytoplasmic DARPP‐32 expression in high and low expression groups of cytoplasmic PP1 and Cdk5 were assessed to understand the impact of these proteins on patient survival. The combination of low DARPP‐32 expression and high PP1 expression was associated with shorter overall survival (*P* < .001; Figure 4A). Low DARPP‐32 and high Cdk5 expression was associated with shorter overall survival (*P* < .001; Figure 4B).

## 4 DISCUSSION

Increasing evidence suggests a role for DARPP‐32 in cancer; however, its role in ovarian cancer remains unclear. This study investigated the expression of DARPP‐32, PP1 and Cdk5 in a cohort of ovarian cancer patients. Low cytoplasmic and nuclear DARPP‐32 expression was associated with shorter overall survival (*P = *.001 and .001, respectively) in the total patient cohort. Importantly, cytoplasmic and nuclear DARPP‐32 expression remained significantly associated with overall survival when other potential confounding factors were included in multivariate analysis (*P* = .001 and .045 respectively). In addition to survival, both nuclear and cytoplasmic DARPP‐32 expression were linked with tumour grade and tumour histology. Limited studies have assessed expression of DARPP‐32 in cancer, with no previous descriptions in ovarian cancer. In breast cancer, low expression of DARPP‐32 was associated with adverse patient survival^9^; in colorectal cancer and glioblastoma multiforme, high expression of DARPP‐32 was associated with adverse survival.^8^, ^32^ In non‐small cell lung and breast cancer, a high t‐DARPP to DARPP‐32 ratio was associated with shorter survival.^4^, ^7^ Studies in murine mammary tumourigenesis suggest that DARPP‐32 is expressed in normal tissue and in some breast tumours, with t‐DARPP expressed only in tumours; this indicates a shift from DARPP‐32 to t‐DARPP expression during tumourigenesis.^10^ Whilst the importance of DARPP‐32, t‐DARPP and the ratio thereof in tumourigenesis remains to be fully understood, evidence in breast cancer implies that DARPP‐32 plays a role in inhibiting cell growth, whilst t‐DARPP accelerates it. In this study, the epitope for the DARPP‐32 antibody used for immunohistochemistry was located within amino acids 0‐30, meaning that DARPP‐32 and not t‐DARPP was assessed.

Expression of PP1 and Cdk5 was assessed in ovarian cancer, with high cytoplasmic and nuclear PP1 expression associated with adverse survival (*P* = .005 and .033, respectively). No association between Cdk5 expression and overall patient survival was observed. Cdk5 expression has been assessed in a number of other tumour types; with low Cdk5 expression associated with adverse survival in gastric cancer^18^ and in breast cancer.^33^ In addition to survival, cytoplasmic PP1 expression was associated with Figo stage, tumour grade, residual disease and histological subtype; with nuclear PP1 expression associated with residual disease and histological subtype. Cytoplasmic Cdk5 expression was associated with Figo stage, residual disease and histological subtype, with nuclear Cdk5 expression associated with tumour grade in addition. These clinicopathological criteria are indicators of poor prognosis and are in line with the associations observed with patient outcome.

In addition to overall survival, we were also able to test associations with progression‐free survival of ovarian cancer patients. Low cytoplasmic and nuclear DARPP‐32 expression, high cytoplasmic PP1 expression and high cytoplasmic Cdk5 expression were associated with shorter progression‐free survival. As none of the proteins were associated with response to chemotherapy this suggests that the association with progression‐free survival is not linked with altered sensitivity to chemotherapy.

Finally, low expression of DARPP‐32 in tumours with high expression of Cdk5 or PP1 was more strongly associated with shorter survival that the alternative combinations. These combined results suggest a loss of DARPP‐32 and/or protein function may be important in ovarian cancer, in particular those with high expression levels of PP1 or Cdk5.

## 5 CONCLUSION

Low cytoplasmic and nuclear DARPP‐32 expression and high cytoplasmic and nuclear PP1 expression are associated with shorter survival in ovarian cancer patients. Importantly, both cytoplasmic and nuclear expression of DARPP‐32 remain associated with overall survival when other confounding factors are included in multivariate analysis. In addition, low cytoplasmic and nuclear DARPP‐32, high cytoplasmic PP1 and high cytoplasmic Cdk5 expression is associated with adverse progression‐free survival. These findings warrant further investigation in larger patient cohorts but indicate that DARPP‐32 expression may be of clinical relevance in ovarian cancer.

## CONFLICT OF INTEREST

The authors declare no conflict of interest.

## AUTHOR CONTRIBUTION

SS, SZ, SY and BS conducted the studies and collected the data; SD provided tissue; SS conducted statistical analysis; SGM and SS conceived the study; SS wrote the manuscript; and all authors approved the manuscript for submission.

## Data Availability

Immunohistochemistry data sets analysed during the current study are available from the corresponding author by request.
